# Bis(3-methyl­anilinium) sulfate

**DOI:** 10.1107/S1600536811045624

**Published:** 2011-11-05

**Authors:** M. Rademeyer

**Affiliations:** a Department of Chemistry, University of Pretoria, Pretoria 0002, South Africa

## Abstract

In the crystal structure of the title salt, 2C_7_H_7_NH_3_^+^·SO_4_^2−^, the cations inter­act with the oxyanions through strong charge-assisted N—H⋯O hydrogen bonds.

## Related literature

The crystal structure of *m*-toluidinium nitrate (Rademeyer & Liles, 2010 ▶), and the structures of three related phosphate salts, namely bis­(*m*-toluidinium) dihydrogen diphosphate (Akriche & Rzaigui, 2000 ▶), tetra­kis­(*m*-toluidinium) cyclo­tetra­phosphate (Aloui *et al.*, 2005 ▶), and hexa­kis­(*m*-toluidin­ium) cyclo­hexa­phosphate (Marouni *et al.*, 2000 ▶), have been reported.  For hydrogen-bond motifs, see: Bernstein *et al.* (1995 ▶). For the most common coordination numbers for the sulfate anion, see: Chertanova & Pascard (1996 ▶).
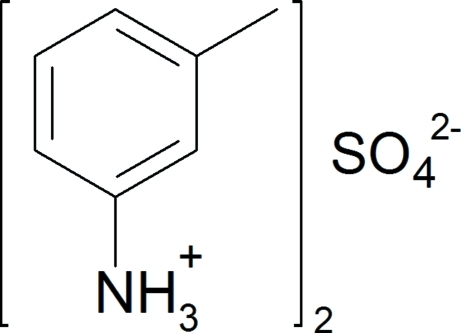


## Experimental

### 

#### Crystal data

2C_7_H_10_N^+^·SO_4_^2−^
*M* *_r_* = 312.23
Monoclinic, 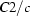

*a* = 17.2168 (8) Å
*b* = 15.0298 (7) Å
*c* = 6.1283 (3) Å
β = 110.819 (3)°
*V* = 1482.25 (12) Å^3^
*Z* = 4
Mo *K*α radiation
μ = 0.24 mm^−1^
*T* = 293 K
0.23 × 0.22 × 0.20 mm

#### Data collection

Oxford Xcalibur2 diffractometer
7603 measured reflections
2404 independent reflections
1615 reflections with *I* > 2σ(*I*)
*R*_int_ = 0.024

#### Refinement

*R*[*F* ^2^ > 2σ(*F* ^2^)] = 0.038
*wR*(*F* ^2^) = 0.135
*S* = 1.04
2404 reflections
104 parameters
H-atom parameters constrained
Δρ_max_ = 0.31 e Å^−3^
Δρ_min_ = −0.27 e Å^−3^

### 

Data collection: *CrysAlis CCD* (Oxford Diffraction, 2006 ▶); cell refinement: *CrysAlis RED* (Oxford Diffraction, 2006 ▶); data reduction: *CrysAlis RED*; program(s) used to solve structure: *SHELXS97* (Sheldrick, 2008 ▶); program(s) used to refine structure: *SHELXL97* (Sheldrick, 2008 ▶); molecular graphics: *Mercury* (Macrae *et al.*, 2006 ▶); software used to prepare material for publication: *PLATON* (Spek, 2009 ▶) and *WinGX* (Farrugia, 1999 ▶).

## Supplementary Material

Crystal structure: contains datablock(s) I, global. DOI: 10.1107/S1600536811045624/bt5698sup1.cif

Structure factors: contains datablock(s) I. DOI: 10.1107/S1600536811045624/bt5698Isup2.hkl

Supplementary material file. DOI: 10.1107/S1600536811045624/bt5698Isup3.cml

Additional supplementary materials:  crystallographic information; 3D view; checkCIF report

## Figures and Tables

**Table 1:** Hydrogen-bond geometry (Å, °)

*D*—H⋯*A*	*D*—H	H⋯*A*	*D*⋯*A*	*D*—H⋯*A*
N1—H1*A*⋯O1	0.89	2.15	2.9384 (19)	147
N1—H1*A*⋯O2	0.89	2.37	3.0913 (18)	139
N1—H1*B*⋯O2^i^	0.89	1.91	2.7997 (18)	173
N1—H1*C*⋯O1^ii^	0.89	1.87	2.7531 (19)	173

Symmetry codes: (i) 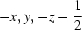; (ii) 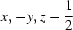.

**Fig. 1.**
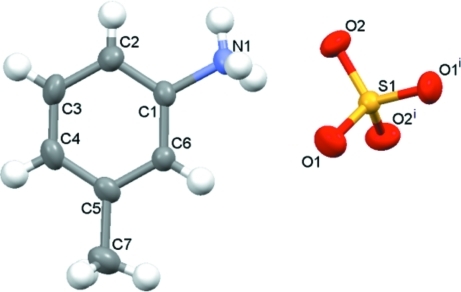
The asymmetric unit of I, showing the atomic numbering scheme. Displacement ellipsoids are shown at the 50% probability level and H atoms are shown as small spheres of arbitrary radii. Symmetry operator i:x, -y, z - 1/2.

**Fig. 2.**
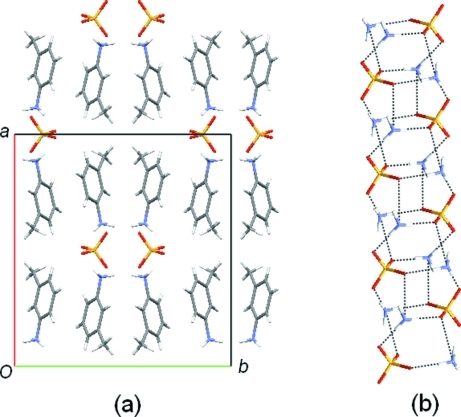
(a) Packing diagram of I viewed down the c-axis. (b) N—H···O hydrogen bonding network in I.

## supplementary crystallographic information

### Comment

Nitrate, sulfate and phosphate anions are important oxyanions in biological processes, the pharmaceutical industry and play a role in freshwater and soil quality. A fundamental understanding of the role of oxyanion geometry on the molecular packing and non-covalent interactions in salt crystal structures is central to the fields of both molecular recognition and crystal engineering.

The molecular geometry and labelling scheme of bis(*m*-toluidinium) sulfate, I, is illustrated in Fig. 1. The asymmetric unit of I consists of one *m*-toluidinium cation and half a sulfate anion, with the S atom on a special position, and the rest of the sulfate anion generated by symmetry. A layered structure, consisting of alternating organic and inorganic layers, is exhibited by I. The organic layers contain the hydrophobic part of the cation, while the inorganic layers comprise the ammonium groups and sulfate anions.

Fig. 2 (*a*) shows the molecular packing of I, viewed down the *c*-axis. Pairs of *m*-toluidinium cations alternate in orientation, and the aromatic groups do not pack in a single row, but forms a sinosoidal wave.

In this structure four cations point to a pair of anions, which places the sulfate anions in a pocket created by ammonium groups. Each sulfate anion accepts six hydrogen bonds from six different cations. This high coordination number indicates the important cohesive role of the sulfate anions in the structure. It has been reported by Chertanova and Pascard (1996) that the most common coordination numbers of the sulfate anion are eight to ten. In I each ammonium group is hydrogen bonded to three different sulfate anions, with hydrogen bonding interactions listed in Table 1. The interactions result in a pseudo-one-dimensional hydrogen bonded ribbon extending along the *c*-direction, which can be described by the graph set notation *R*_4_^4^(12) (Bernstein, 1995). Hydrogen bonding interactions are illustrated in Fig. 2 (*b*). Pairs of cations interact through aromatic interactions in a slipped, head-to-tail fashion, with a centroid-to-centroid distance of 3.6025 (9) Å. Planes through neigbouring cation pairs intersect at an angle of 58°.

### Experimental

Bis(*m*-toluidinium) sulfate was prepared by the dropwise addition of excess concentrated sulfuric acid (0.35 ml, 98%, Aldrich) to a solution of *m*-toluidine (0.50 ml, 99%, Aldrich) in 20 ml chloroform (99%, Saarchem). The resulting precipitate was filtered, dried in air and re-crystallized from distilled water. Colourless crystals formed on evaporation, open to the air, at room temperature.

### Refinement

All H atoms were refined using a riding model, with C—H distances either 0.93 or 0.96 Å and N—H distances of 0.89 Å, and *U*_iso_(H) = 1.5*U*_eq_(C) or 1.2*U*_eq_(C) or 1.2*U*_eq_(N). The highest residual peak is 0.71 Å from atom O2.

### Crystal data

**Table:**

2C_7_H_10_N^+^·SO_4_^2^^−^	*F*(000) = 664
*M**_r_* = 312.23	*D*_x_ = 1.399 Mg m^−^^3^
Monoclinic, *C*2/*c*	Mo *K*α radiation, λ = 0.71073 Å
Hall symbol: -C 2yc	Cell parameters from 3393 reflections
*a* = 17.2168 (8) Å	θ = 3.6–32.1°
*b* = 15.0298 (7) Å	µ = 0.24 mm^−^^1^
*c* = 6.1283 (3) Å	*T* = 293 K
β = 110.819 (3)°	Block, colourless
*V* = 1482.25 (12) Å^3^	0.23 × 0.22 × 0.20 mm
*Z* = 4	

### Data collection

**Table:**

Oxford Xcalibur2 diffractometer	1615 reflections with *I* > 2σ(*I*)
Radiation source: fine-focus sealed tube	*R*_int_ = 0.024
graphite	θ_max_ = 32.1°, θ_min_ = 3.6°
ω–2θ scans	*h* = −24→24
7603 measured reflections	*k* = −21→20
2404 independent reflections	*l* = −8→8

### Refinement

**Table:**

Refinement on *F*^2^	Primary atom site location: structure-invariant direct methods
Least-squares matrix: full	Secondary atom site location: difference Fourier map
*R*[*F*^2^ > 2σ(*F*^2^)] = 0.038	Hydrogen site location: inferred from neighbouring sites
*wR*(*F*^2^) = 0.135	H-atom parameters constrained
*S* = 1.04	*w* = 1/[σ^2^(*F*_o_^2^) + (0.0795*P*)^2^] where *P* = (*F*_o_^2^ + 2*F*_c_^2^)/3
2404 reflections	(Δ/σ)_max_ = 0.017
104 parameters	Δρ_max_ = 0.31 e Å^−^^3^
0 restraints	Δρ_min_ = −0.27 e Å^−^^3^

### Special details

**Table:**

Geometry. All e.s.d.'s (except the e.s.d. in the dihedral angle between two l.s. planes) are estimated using the full covariance matrix. The cell e.s.d.'s are taken into account individually in the estimation of e.s.d.'s in distances, angles and torsion angles; correlations between e.s.d.'s in cell parameters are only used when they are defined by crystal symmetry. An approximate (isotropic) treatment of cell e.s.d.'s is used for estimating e.s.d.'s involving l.s. planes.
Refinement. Refinement of *F*^2^ against ALL reflections. The weighted *R*-factor *wR* and goodness of fit *S* are based on *F*^2^, conventional *R*-factors *R* are based on *F*, with *F* set to zero for negative *F*^2^. The threshold expression of *F*^2^ > σ(*F*^2^) is used only for calculating *R*-factors(gt) *etc*. and is not relevant to the choice of reflections for refinement. *R*-factors based on *F*^2^ are statistically about twice as large as those based on *F*, and *R*- factors based on ALL data will be even larger.

### Fractional atomic coordinates and isotropic or equivalent isotropic displacement parameters (Å^2^)

**Table:**

	*x*	*y*	*z*	*U*_iso_*/*U*_eq_	
N1	0.11567 (8)	0.10113 (10)	−0.1357 (2)	0.0333 (3)	
H1A	0.1002	0.1171	−0.0174	0.053 (6)*	
H1B	0.0868	0.1321	−0.2624	0.057 (6)*	
H1C	0.1061	0.0433	−0.1641	0.054 (6)*	
C1	0.20457 (9)	0.11906 (9)	−0.0750 (3)	0.0280 (3)	
C2	0.23035 (10)	0.16302 (11)	−0.2339 (3)	0.0361 (4)	
H2	0.1928	0.1786	−0.3801	0.043 (5)*	
C3	0.31438 (10)	0.18371 (12)	−0.1699 (3)	0.0417 (4)	
H3	0.3332	0.2138	−0.2741	0.081 (7)*	
C4	0.36970 (10)	0.15994 (11)	0.0457 (3)	0.0385 (4)	
H4	0.4255	0.1750	0.0863	0.066 (7)*	
C5	0.34354 (9)	0.11370 (10)	0.2051 (3)	0.0325 (3)	
C6	0.25958 (9)	0.09370 (10)	0.1415 (3)	0.0305 (3)	
H6	0.2404	0.0633	0.2445	0.038 (5)*	
C7	0.40487 (11)	0.08698 (13)	0.4386 (3)	0.0490 (5)	
H7A	0.3758	0.0615	0.5313	0.073*	
H7B	0.4353	0.1384	0.5165	0.073*	
H7C	0.4428	0.0439	0.4174	0.073*	
S1	0.0000	0.13146 (3)	0.2500	0.02700 (17)	
O1	0.07354 (8)	0.07574 (8)	0.2846 (2)	0.0472 (3)	
O2	−0.01386 (7)	0.18692 (7)	0.04230 (19)	0.0432 (3)	

### Atomic displacement parameters (Å^2^)

**Table:**

	*U*^11^	*U*^22^	*U*^33^	*U*^12^	*U*^13^	*U*^23^
N1	0.0271 (6)	0.0429 (8)	0.0312 (7)	−0.0030 (6)	0.0119 (5)	−0.0038 (6)
C1	0.0257 (7)	0.0288 (7)	0.0318 (7)	0.0002 (6)	0.0130 (6)	−0.0036 (6)
C2	0.0374 (8)	0.0407 (9)	0.0325 (9)	0.0031 (7)	0.0154 (7)	0.0034 (7)
C3	0.0415 (9)	0.0435 (10)	0.0493 (10)	−0.0004 (7)	0.0274 (8)	0.0087 (7)
C4	0.0282 (7)	0.0360 (8)	0.0545 (10)	0.0014 (6)	0.0186 (7)	0.0015 (7)
C5	0.0271 (7)	0.0273 (7)	0.0408 (9)	0.0052 (6)	0.0093 (6)	0.0007 (6)
C6	0.0296 (7)	0.0293 (7)	0.0338 (8)	0.0000 (6)	0.0127 (6)	0.0040 (6)
C7	0.0365 (9)	0.0502 (11)	0.0515 (12)	0.0048 (8)	0.0049 (8)	0.0068 (8)
S1	0.0270 (3)	0.0297 (3)	0.0256 (3)	0.000	0.0108 (2)	0.000
O1	0.0413 (7)	0.0497 (7)	0.0526 (8)	0.0160 (6)	0.0190 (6)	−0.0040 (6)
O2	0.0554 (8)	0.0420 (7)	0.0309 (6)	−0.0104 (6)	0.0137 (5)	0.0067 (5)

### Geometric parameters (Å, °)

**Table:**

N1—C1	1.4659 (17)	C4—H4	0.9299
N1—H1A	0.8899	C5—C6	1.390 (2)
N1—H1B	0.8901	C5—C7	1.500 (2)
N1—H1C	0.8899	C6—H6	0.9299
C1—C2	1.374 (2)	C7—H7A	0.9600
C1—C6	1.382 (2)	C7—H7B	0.9600
C2—C3	1.393 (2)	C7—H7C	0.9600
C2—H2	0.9300	S1—O2^i^	1.4684 (11)
C3—C4	1.373 (2)	S1—O2	1.4684 (11)
C3—H3	0.9300	S1—O1^i^	1.4692 (12)
C4—C5	1.397 (2)	S1—O1	1.4692 (12)
			
C1—N1—H1A	109.4	C6—C5—C4	118.22 (15)
C1—N1—H1B	109.5	C6—C5—C7	121.27 (15)
H1A—N1—H1B	109.5	C4—C5—C7	120.50 (15)
C1—N1—H1C	109.5	C1—C6—C5	119.97 (14)
H1A—N1—H1C	109.5	C1—C6—H6	120.0
H1B—N1—H1C	109.5	C5—C6—H6	120.0
C2—C1—C6	121.94 (14)	C5—C7—H7A	109.5
C2—C1—N1	118.66 (14)	C5—C7—H7B	109.5
C6—C1—N1	119.37 (13)	H7A—C7—H7B	109.5
C1—C2—C3	118.21 (15)	C5—C7—H7C	109.5
C1—C2—H2	121.0	H7A—C7—H7C	109.5
C3—C2—H2	120.8	H7B—C7—H7C	109.5
C4—C3—C2	120.56 (15)	O2^i^—S1—O2	110.82 (9)
C4—C3—H3	119.7	O2^i^—S1—O1^i^	108.34 (7)
C2—C3—H3	119.8	O2—S1—O1^i^	109.42 (7)
C3—C4—C5	121.08 (15)	O2^i^—S1—O1	109.42 (7)
C3—C4—H4	119.4	O2—S1—O1	108.34 (7)
C5—C4—H4	119.5	O1^i^—S1—O1	110.51 (11)
			
C6—C1—C2—C3	−1.3 (2)	C3—C4—C5—C7	179.07 (17)
N1—C1—C2—C3	176.82 (14)	C2—C1—C6—C5	0.9 (2)
C1—C2—C3—C4	0.5 (3)	N1—C1—C6—C5	−177.23 (13)
C2—C3—C4—C5	0.7 (3)	C4—C5—C6—C1	0.3 (2)
C3—C4—C5—C6	−1.2 (3)	C7—C5—C6—C1	−179.89 (15)

Symmetry codes: (i) −*x*, *y*, −*z*+1/2.

### Hydrogen-bond geometry (Å, °)

**Table:**

*D*—H···*A*	*D*—H	H···*A*	*D*···*A*	*D*—H···*A*
N1—H1A···O1	0.89	2.15	2.9384 (19)	147.
N1—H1A···O2	0.89	2.37	3.0913 (18)	139.
N1—H1B···O2^ii^	0.89	1.91	2.7997 (18)	173.
N1—H1C···O1^iii^	0.89	1.87	2.7531 (19)	173.

Symmetry codes: (ii) −*x*, *y*, −*z*−1/2; (iii) *x*, −*y*, *z*−1/2.
